# Antimitochondrial effects of thioacetamide and ethylenethiourea in human and yeast cell cultures

**DOI:** 10.1038/bjc.1980.209

**Published:** 1980-07

**Authors:** E. Diala, U. Mittwoch, D. Wilkie

## Abstract

**Images:**


Br. J. Cancer (1980) 42, 112

### ANTIMITOCHONDRIAL EFFECTS OF THIOACETAMIDE AND ETHYLENETHIOUREA IN HUMAN AND YEAST CELL CULTURES

E. DIALA*, U. MITTWOCH* AND D. WILKIE†

*From the Departments of *Genetics and Biometry, †Botany and Microbiology, University College London, Gower Street, London WC1E 6BT*

Received 14 November 1979 Accepted 14 March 1980

**Summary. Cytological studies in the light microscope showed that thioacetamide (TAA) depressed the mitotic index in cultures of skin fibroblasts at the lowest concentrations used (100 μg/ml). At high concentration (1 mg/ml), TAA tended to cause aberration in nuclear morphology. Ethylenethiourea (ETU) had no effect on either mitotic index or nuclear morphology at 1 mg/ml. Fibroblast cultures treated with 1 mg/ml TAA and cultures grown in the presence of 2 mg/ml ETU were studied by electron microscopy. In some TAA-treated cells there was unfolding of the nuclear membrane and enlargement and granulation of the nucleolus, but these effects were not correlated. In all cells, TAA caused severe and characteristic damage to the majority of mitochondria, whether or not there were nuclear aberrations. The organelle showed extensive swelling of the cristae of the inner membrane and an increase in matrix density. Ultrastructure of other cell components appeared to be unaffected by this treatment. In ETU-treated cells some less severe swelling of inner mitochondrial membranes was seen and only in a minority of cells, whilst all other cell structures appeared normal. Similar membrane swelling and increase in matrix density was seen in isolated rat liver mitochondria after incubation with TAA, indicating a direct antimitochondrial effect of the carcinogen.**

**When yeast cells were treated with TAA and ETU, primary antimitochondrial activity of these compounds was apparent from (1) inhibition of growth in non-fermentable medium, (2) selective blockage of mitochondrial protein synthesis and (3) induction of mitochondrial mutations. TAA was much more effective than ETU in all these respects.**

THIOACETAMIDE (TAA) and ethylenethiourea (ETU) have similarities in their structural formulae (Fig. 1) but, whereas TAA readily induces hepatomas in rats (Fitzburgh & Nelson, 1948), ETU is claimed to be tumorigenic only after prolonged treatment at high doses (Ulland *et al.*, 1972). Preliminary results indicated that for a number of carcinogens, including TAA, mitochondria are the primary targets in yeast cells (Egilsson *et al.*, 1979). These findings raise two questions: does the antimitochondrial activity of TAA and other carcinogens extend to human cells and, if so, is the severity of the antimitochondrial effect related to carcinogenic potency?

FIG. 1.—Chemical structure of ethylenethiourea (i) and thioacetamide (ii).

We have attempted to answer both questions by investigating the ultrastructure of human diploid fibroblasts treated with TAA and ETU. The results provide *prima facie* evidence that mitochondria

† To whom requests for reprints should be addressed.

are affected, and more by TAA than by ETU.

To corroborate these findings, the same compounds have been studied in the yeast system, in which primary antimitochondrial activity is detectable by several techniques not available for human cells (Wilkie, 1972). Briefly, the test exploits the ability of yeast cells to dispense with mitochondrial respiration (*e.g.* under anaerobiosis) and grow on the products of glycolysis. Thus a compound which selectively or specifically inhibits mitochondria will have little or no effect on yeast growth provided glucose is available. The same compound would arrest growth if the carbon source was non-fermentable (*e.g.* glycerol) requiring a functional respiratory chain to be metabolized. The initial step, then, in establishing the primary mitochondrial activity of a drug is the arrest of growth in glycerol medium at concentrations of the drug which permit growth in glucose medium.

Antimitochondrial activity in the case of a carcinogen or mutagen would be expected to result from direct or indirect reaction with mitochondrial DNA, which could then be affected both in its transcription and replication. The former effect is detectable as failure of cytochromes $aa_3$ and $b$ to develop, since the appearance of these respiratory-chain enzymes requires synthesis in the mitochondria of mitochondrially coded polypeptides. Cytochrome $c$, on the other hand, is coded by a nuclear gene and synthesized on cytoplasmic ribosomes and so would be largely unaffected by the mitochondrial inhibitor. These effects are readily scorable from the cytochrome absorption spectra using whole yeast cells (Fig. 5).

If mitochondrial DNA replication is disturbed, genetic lesions (deletions) in this molecule will readily occur giving rise to respiratory deficiency recognized as *petite* colonies from platings on glucose medium. The *petite* condition arises spontaneously with a high frequency (usually 1%) resulting from extensive deletions of mitochondrial DNA. This high frequency distinguishes this category of respiratory mutant from nuclear mutations. All *petites* appear to be irreversible and none can carry out mitochondrial protein synthesis. Thus absorption spectra show the presence of cytochrome $c$ and absence of $aa_3$ and $b$. For further information on the *petite* mutation, see the review of Bernardi (1979).

### MATERIALS AND METHODS

*Human cell cultures.*—All experiments were carried out on human skin fibroblasts with a normal diploid karyotype. None of the cells had undergone more than 20 culture passages. The donors of the cells were one male child, aged 2·5 years (A), one male stillborn infant (B), and a female foetus (C).

Cell cultures were maintained in Eagle's Minimum Essential Medium (Flow Laboratories Ltd) supplemented with 10% foetal calf serum, 10 mM HEPES buffer, penicillin (100 iu/ml) and streptomycin (100 $\mu$g/ml).

For mitotic counts, the cells were trypsinized (0·15% trypsin in Versene) and resuspended in growth medium containing 20% human serum instead of 10% foetal calf serum, at a concentration of $10^5$ cells/ml. Aliquots of 1·5 ml of this suspension were introduced into 30mm plastic Nunclon Petri dishes containing 1 coverslip each, 1·5 ml of suspension being placed in each dish. TAA and ETU stock solutions were made by dissolving the compounds in growth medium which was then Seitz-filtered (0·22$\mu$m Millipore filter discs). Appropriate amounts of medium containing the carcinogens to give the required final concentration were added to the dishes at the time of plating, and equal volumes of Seitz-filtered growth media were added to the control dishes.

Partially synchronized cultures were obtained by trypsinizing confluent cultures and re-seeding at lower cell densities. At the completion of treatment, cover slips were removed and the cells were fixed in 95% ethanol and stained by the Feulgen procedure. Mitoses were scored using an oil-immersion objective ( × 100) and an eyepiece graticule (Modified Whipple, Graticules Ltd). TAA was obtained from Sigma Chemical Co. and ETU from Rohm & Haas Co., Philadelphia, Pennsylvania, U.S.A.

*Electron microscopy.*—Cultures were prepared by inoculating 1·5 ml of suspensions containing $10^5$ cells/ml into Nunclon Petri dishes. TAA and ETU were added to the culture medium from the Seitz-filtered stock solutions. After an incubation period of 48 h, at 37°C, the cells were fixed at room temperature, and embedded *in situ*, according to the method of Brinkley *et al.* (1967) with the following modifications: monolayers were washed in Dulbecco A (3) NaCl solution, fixed in 4% paraformaldehyde/glutaraldehyde (in 0·1M sodium cacodylate, $Na(CH_3)_2$-$AsO_2.3H_2O$, and 0·25M sucrose, pH 7·4), washed ×6 for 5 min each in Dulbecco A solution, and finally, post-fixed in 1% $OsO_2$ (in 0·1M sodium cacodylate, 0·25M sucrose, pH 7·4) for 1 h at room temperature. The cultures were then rapidly dehydrated in 35% ethanol for 1 min, soaked in a 60% ethanol/40% resin mixture (EMix resin, EMscopes Laboratories Ltd, Kent) for 5 min, transferred to a 30% ethanol/70% resin mixture for a further 5 min, infiltrated in pure resin for 5 min and, finally, re-infiltrated in another fresh change of pure resin and polymerized for 24 h at 60°C.

Thin sections (60–90 nm) were examined in an AEI-801 Electron Microscope, at an acceleration voltage of 60 kV.

*Yeast cultures.*—18 strains of the yeast *Saccharomyces cerevisiae* from the collection of this laboratory were used. Culture media contained 1% Difco yeast extract and either 2% D-glucose (YED) or 4% glycerol (YEG) as carbon source. 2% Difco bacto-agar was used to solidify medium where required. TAA and ETU were added to autoclaved medium from stock solutions. In growth tests, a multiple inoculation procedure was used (Wilkie, 1972).

*Absorption spectra.*—Cultures were grown in shake culture in YED medium to stationary phase in the presence and absence of TAA and ETU. Cells were harvested by centrifugation, washed twice in ice-cold distilled water and resuspended as thick slurry (~ $^9 10q$ cells/ml) also in distilled water. Absorption spectra in the 500nm–630nm region were scanned using a Pye-Unicam SPI800 recording spectrophotometer at room temperature, with a tissue-paper blank.

Petite *mutation.* — *Petite* colonies were scored initially by size and colour on YED agar, subsequently by velvet-pad transfer to YEG medium where they failed to grow.

*Rat liver mitochondria.*—Mitochondria were prepared according to Chappell & Hunsford (1972) and incubated as described by Wallis & Wilkie (1979) for 3 h in the presence and absence of 20mM TAA. Mitochondria were then prepared for EM study following the method of Diala (1978).

### RESULTS

### *Human cells*

The effects of TAA and ETU on cell division are recorded in Table I. ETU, up to 2 mg/ml in the culture medium, inhibited effect on growth and division of fibroblasts. On the other hand, TAA at concentrations of 100 and 250 μg/ml depressed the mitotic rate, while in Exp. 2, 1 mg/ml totally blocked cell division and also caused severe effects on the nucleus, which showed marked convolutions similar to those described in tumour cells of malignant melanoma by Hunter *et al.* (1978) (Fig. 2c). In Exp. 4, treatment with 1 mg/ml TAA did not produce this morphological aberration, but some inhibition of mitosis was still apparent (Table I).

Seven further experiments were carried out in which the effects of TAA (0·5 and 1 mg/ml) and ETU (1 and 2 mg/ml) on morphology were directly compared in cells from the different donors (A, B and C: see Table I) and under varying con-

TABLE I.—*Effect of thioacetamide (TAA) and ethylenethiourea (ETU) on cell division in human cell cultures*

| Exp. | Cell line | Treatment | Number in division* per 5000 cells |
|---|---|---|---|
| 1 | A | ETU | |
| | | 1000 μg/ml | 67 |
| | | 100 μg/ml | 54 |
| | | Control | 61 |
| 2 | A | TAA | |
| | | 1000 μg/ml | 0 |
| | | 100 μg/ml | 1 |
| | | Control | 22 |
| 3 | A | TAA | |
| | | 250 μg/ml | 62 |
| | | 100 μg/ml | 126 |
| | | Control | 142 |
| 4 | C | TAA | |
| | | 1000 μg/ml | 14 |
| | | ETU | |
| | | 2000 μg/ml | 30 |
| | | Control | 37 |

* Cells observed 25–38 h after plating.

ditions. These included time of exposure, passage number, and type of serum (20% human *vs* 10% foetal calf) in the culture medium. No abnormalities were seen after treatment with ETU in any of these cultures, but cells from A, B and C treated with 1 mg/ml TAA showed cellular and nuclear contortion, though cells were probably not killed by this treatment, since they remained attached to the cover-slip. Treatment with 0·5 mg/ml TAA produced no apparent morphological disturbance. Thus the 3 different cell lines had similar responses to the various treatments.

*Electron microscopy*

A high proportion of the mitochondria in all cells of culture A treated with 1 mg/ml TAA showed structural aberrations, characterized by a dense matrix and excessive swelling of the inner membrane (Fig. 3b). In contrast, ETU-treated cells showed fewer cases of structural aberration in the mitochondria, which were mostly indistinguishable from mitochondria of the control cells (Fig. 3c). No other structural damage was detected in ETU cultures. The aberration seen in mitochondria was mainly inner-membrane swelling, but this was not as pronounced as in the TAA-treated cells. Although these observations were made using only one human cell line and at selected concentrations of TAA and ETU in the growth medium, the results were clear-cut and widely different for the two compounds. Results obtained with cells from different donors in the light-microscope studies justify the extrapolation to other lines.

Nuclear contortion in TAA-treated cells was recognizable in EM as extensive folding of the nuclear membrane (Fig. 2c). The aberration was seen only in some of the cells. A main point is that in those cells with no detectable nuclear distortion the mitochondrial effect was still much in evidence (Fig. 2d). At the same time, other membrane structures (including Golgi, microtubules and microfilaments, lysosomes and endoplasmic reticulum) and overall cellular morphology appeared unaffected. On the other hand, the nucleolus tended to be altered in a characteristic way in these cells, being enlarged with a distinctive increase in granular elements compared with the normal nucleolus (Fig. 2c). Nuclear substructure in ETU-treated cells appeared normal. More than 200 nuclei were examined in these studies. It has been reported that the nucleoli of rat liver cells treated *in vivo* with TAA likewise showed a marked increase in the granular component (Svoboda & Higginson, 1968) a condition thought to be due to accumulation of RNA in the nucleoli. At the same time, mitochondria in these preparations showed an increased matrix density and poorly developed cristae. It was possible in our preparations to see an apparently normal nucleolus in a cell showing the typical mitochondrial aberration induced by TAA, which suggests the primary nature of this effect (Fig. 2d).

*Rat liver mitochondria*

As can be seen in Fig. 4, TAA caused mitochondrial aberrations similar to those of intact cells, namely swollen and distended membranes (note fragile condition) with a dense matrix. These findings demonstrated that TAA could directly affect mitochondrial structure.

*Yeast system*

In all strains tested, both TAA and ETU were more inhibitory to growth in glycerol medium than in medium with glucose as carbon source, indicating primary antimitochondrial action of both compounds. The minimum inhibitory concentration (MIC) of ETU in glycerol medium was, on average, $\sim 1$ mg/ml, whereas that of TAA was $\sim 200$ $\mu$g/ml. General cytotoxicity of TAA was indicated by inhibition of growth in glucose medium at a concentration of 1 mg/ml, but ETU at maximum solubility (4 mg/ml) did not arrest growth in glucose, though the rate of growth was affected in some strains. The results indicated that both compounds selectively blocked mitochondria and that

(a) (b)

(c)

(d)

Fig. 2.—(a, b) Photo micrographs of human fibroblast nuclei (48 h) Feulgen stain, × 1845. (a) control cell, (b) 2 cells after treatment with 1 mg/ml TAA, showing distorted nuclei. (c, d) Thin-section EM micrographs of 1 mg/ml TAA-treated fibroblast cells grown for 48 h. (c) unusual coiling of the nuclear membrane (nm) and the dense, granular nucleolus (nc), × 10,760; (d) typical swollen mitochrondrion (m) but apparently normal nuclear structures, × 15,380. Rough endoplasmic reticulum, rER; Golgi, G; lysosome, L; microfilaments, MF.

(a)

(b)

(c)

FIG. 3.—EM comparison of human fibroblast mitochondria. a, control, × 41,700; b, TAA-treated (1 mg/ml), × 45,800 (the inner mitochondrial membranes are swollen and the mitochondrial matrix is very dense, relative to the control); c, ETU-treated (2 mg/ml), × 41,700 (slight swelling of membrane cristae, but not grossly different from control).

FIG. 4.—Effect of thioacetamide (TAA) on the ultrastructure of isolated rat liver mitochondria. (See Materials and Methods for procedure.) a, untreated, × 32,000; b, TAA-treated, × 28,800; c, TAA-treated, × 58,400. Note distended and fragile condition of treated mitochondria and compare with Fig. 3b.

TAA was more active than ETU in this respect.

Selective blockage of mitochondria was further demonstrated by failure of cytochromes $aa_3$ and $b$ to develop during growth in glucose medium in the presence of the two compounds (Fig. 5). Thus mitochondrial protein synthesis was arrested while cytoplasmic protein synthesis, exemplified by the appearance of cytochrome $c$, continued.

Interaction with mitochondrial DNA was indicated by these compounds in the induction of *petite* mutants (Table II). Induction by TAA was extensive but the mutagenic activity of ETU was slight. It was shown that the increased frequency of *petite* mutants in treated cultures was induction and not selection, by micromanipulating cells of *petite* mutants on to agar blocks containing TAA and ETU. It was found that the rate of division of

FIG. 5.—Absorption spectra of whole yeast cells, haploid Strain D4, grown to stationary phase in 1% yeast extract, 2% glucose medium in the absence of inhibitor (a); in the presence of 0·1 mg/ml TAA (b); 0·5 mg/ml ETU (c); and, 2 mg/ml ETU (d).

The cytochrome absorbing region ranges from 500 nm to 630 nm. The vertical bar represents 0·1 OD unit and the major absorption peaks for cytochromes $aa_3$, *b*, and *c* are, respectively, 603 nm, 562 nm and 552 nm. β peaks of cytochromes *c* and *b* are seen at 521 nm and 532 nm respectively.

TABLE II.—*Mitochondrial mutagenesis by thioacetamide (TAA) and ethylenethiourea (ETU) in* Saccharomyces cerevisiae*: induction of* petite *colony*

| Compound | Concentration (mg/ml) | *Petite* colonies | Total colonies | % *petite* |
|---|---|---|---|---|
| TAA | 0·2 | 178 | 622 | 28·6 |
| ETU | 0·5 | 15 | 560 | 2·7 |
| | 2·0 | 38 | 1180 | 3·2 |
| Control | — | 19 | 1110 | 1·6 |

Results listed are for Laboratory Strain D6, but essentially similar results were obtained ni 3 other strains: B21, D22 and B41.

mutant cells was not faster than that of normal cells but was, in fact, slightly slower in most cases in the presence of these compounds. Taken together, these results are strong evidence of selective inhibition of mitochondrial biogenesis.

### DISCUSSION

The tentative conclusion that TAA selectively affects mitochondrial development in human cells is based on the EM observations that ultrastructural damage to the organelle was (1) characteristic and occurred in all cells treated with TAA and (2) observed in cells where other structures appeared normal (applies also to ETU). However, these observations in themselves do not provide conclusive evidence of primary antimitochondrial activity, and it could be argued that the mitochondrial effects were secondary to more general toxicity of TAA. To prove or disprove this point would be extremely difficult in this system.

The results with yeast cells were less equivocal and clearly indicated a selective reaction by TAA (and to a lesser extent ETU) with mitochondrial DNA, causing mutations and affecting biogenesis. Whether these yeast results support direct mitochondrial effects in human cells depends on how readily results from one eukaryotic cell type can be extrapolated to another. In view of the striking similarities in function, structure, organization and reproductive characteristics of all mitochondria, yeast and human (even to intrinsic deviations from the accepted rules in triplet coding) some extrapolation would appear to be justified. No mitochondrial poison is known to affect yeast and not mammalian mitochondria.

The possibility of carcinogens selectively reacting with mitochondrial DNA was raised by Graffi and associated (Wunderlich *et al.*, 1970) who found that N-methyl-N-nitrosourea and nitrosodimethylamine selectively alkylated mitochondrial DNA rather than nuclear DNA in animals administered these carcinogens. Carcinogenic azo dyes have also been found to have a preferential affinity for cytoplasmic structures in mammalian cells (Nagatani, 1960). The sensitivity of mitochondrial DNA to alkylating agents and other DNA-reacting compounds may be due to the unprotected nature of this molecule; it is not complexed with packaging proteins as is nuclear DNA but is a naked, circular molecule.

This circularity may also be a source of vulnerability, if the induction of sister-chromatid exchange, a well established activity of chemical carcinogens in replicating mammalian chromosomes (Wolff, 1977) is taken into account. Exchange between circular chromosomes could introduce mechanical problems, leading to catenated circles and/or large circles containing multiple genomes. Indeed it is claimed that catenated and other aberrant mitochondrial DNA molecules are a feature of certain tumour cells (Clayton & Smith, 1975). Whether TAA and ETU primarily react (directly or indirectly) with mitochondrial DNA of human cells remains an open question, though our results are suggestive.

The possible connection between primary mitochondrial change and oncogenesis is discussed by Egilsson *et al.* (1979) and is based on our findings that mitochondrial mutagenesis by chemical carcinogens has a pleiotropic effect on cell-surface characteristics in yeast cells.

This investigation was supported by Cancer Research Campaign grants to U. Mittwoch and D. Wilkie. Excellent technical assistance by Mrs S. Mahadevaiah and Mrs D. Collier is gratefully acknowledged. We also wish to thank Mr G. Lawes for expert assistance at the EM facility, Birkbeck College, University of London.

### REFERENCES

BERNARDI, G. (1979) The *petite* mutation in yeast. *Trends Bid. Sci.*, **4**, 197.

BRINKLEY, B. R., MURPHY, P. & RICHARDSON, L. C. (1967) Procedure for embedding *in situ* selected cells cultured *in vitro. J. Cell Biol.*, **35**, 279.

CHAPPELL, J. B. & HUNSFORD, R. G. (1972) *Subcellular Components, Preparation and Fractionation.* London: Butterworths. p. 000.

CLAYTON, D. A. & SMITH, C. A. (1975) Complex mitochondrial DNA. In *International Review of Experimental Pathology.* Eds Richter & Epstein. New York: Academic Press. p. 125.

DIALA, E. S. (1978) Ph.D. thesis (Univ. of London).

DULBECCO, R. & VOGT, M. (1954) Plaque formation and isolation of pure lines with poliomyelitis viruses. *J. Exp. Med.*, **99**, 167.

EGILSSON, V., EVANS, I. H. & WILKIE, D. (1979) Toxic and mutagenic effects of carcinogens on the mitochondria of *Saccharomyces cerevisiae. Mol. Gen. Genet.*, **174**, 39.

FITZBURGH, O. G. & NELSON, A. A. (1948) Liver tumours in rats fed thiourea and thioacetamide. *Science*, **108**, 626.

HUNTER, J. A., ZAYNOUN, S., PATERSON, W. D., BLEEHEN, S. S., MACKIE, R. & COCKRAN, A. J. (1978) Cellular fine structre in the invasive nodules of different histogenetic types of malignant melanoma. *Br. J. Dermatol.*, **98**, 255.

NAGATANI, Y. (1960) Cytological studies on azo dyes. *Int. Rev. Cytol.*, **10**, 243.

SVOBODA, D. & HIGGINSON, J. (1968) A comparison of ultrastructural changes in rat liver due to chemical carcinogens. *Cancer Res.*, **28**, 1703.

ULLAND, B. M., WEISBURGER, J. H., WEISBURGER, E. K., RICE, J. M. & CYPHER, R. (1972) Thyroid cancer in rats from ethylenethiourea uptake. *J. Natl Cancer Inst.*, **49**, 563.

WALLIS, C. & WILKIE, D. (1979) Mitochondrial activity of 2-6 diaminopurine in *Saccharomyces cerevisiae. Mol. Gen. Genet.*, **173**, 307.

WILKIE, D. (1972) The yeast cell in antimitochondrial activity of drugs. *Med. Biol. Illus.*, **22**, 119.

WOLFF, S. (1977) Sister chromatid exchange. *Ann. Rev. Genet.*, **11**, 183.

WUNDERLICH, V., SCHUTT, M., BOTTGER, M. & GRAFFI, A. (1970) Preferential alkylation of mitochondrial DNA by N-methyl-nitrosourea. *Biochem. J.*, **118**, 99.


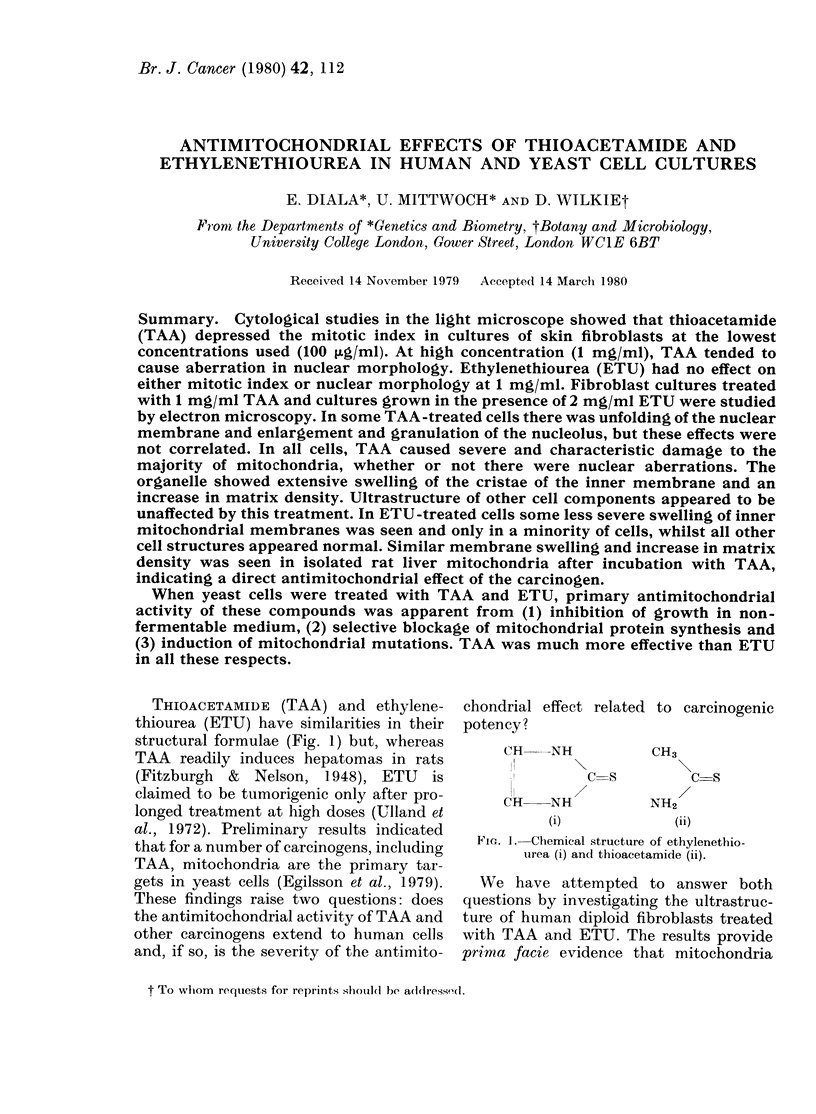

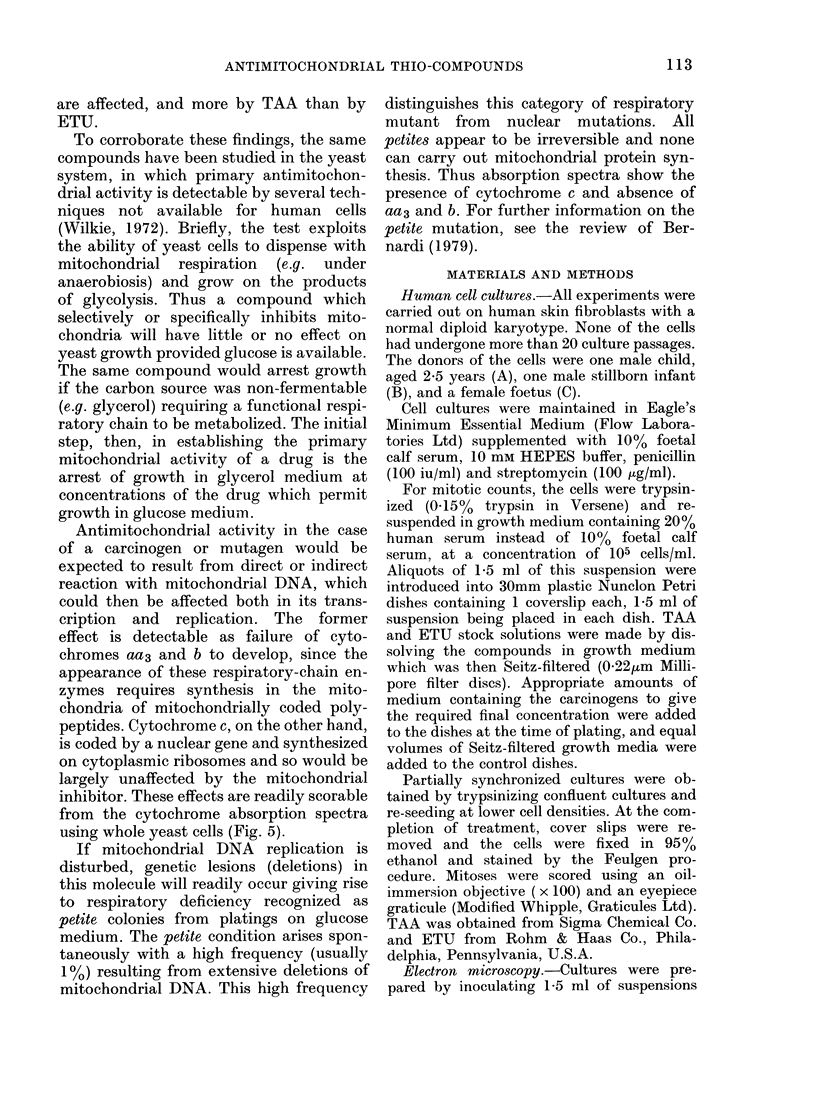

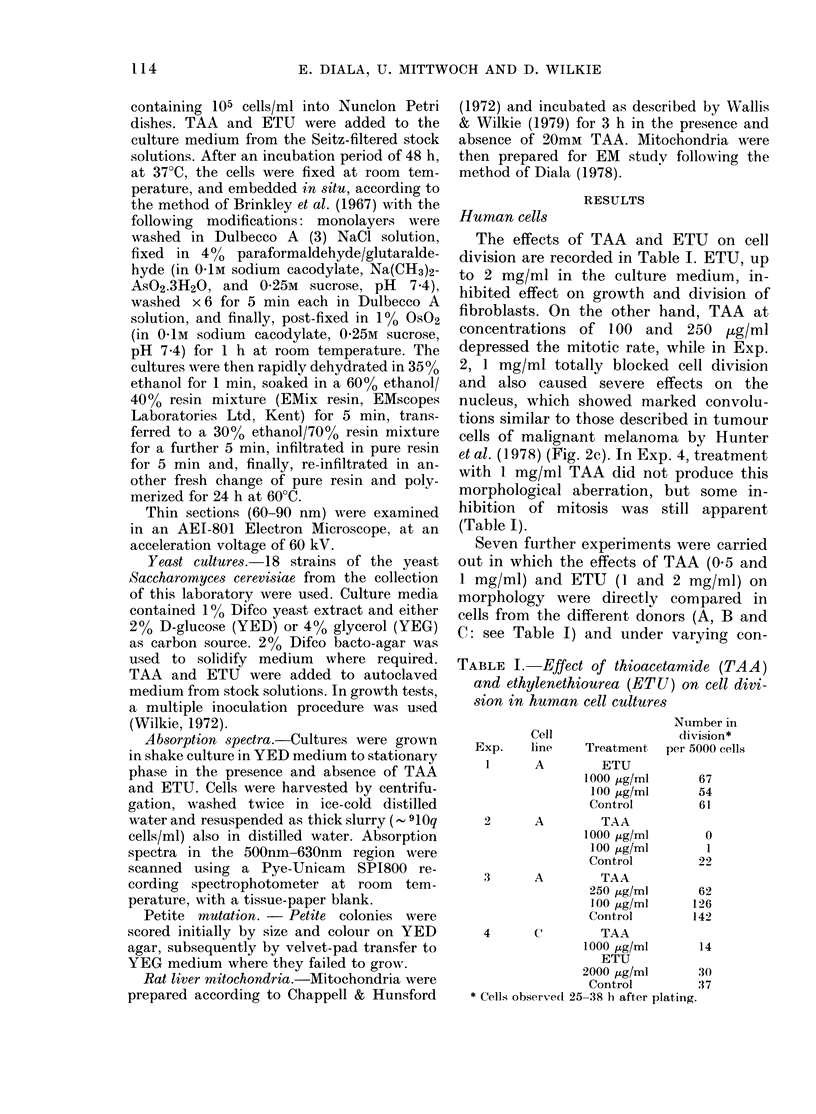

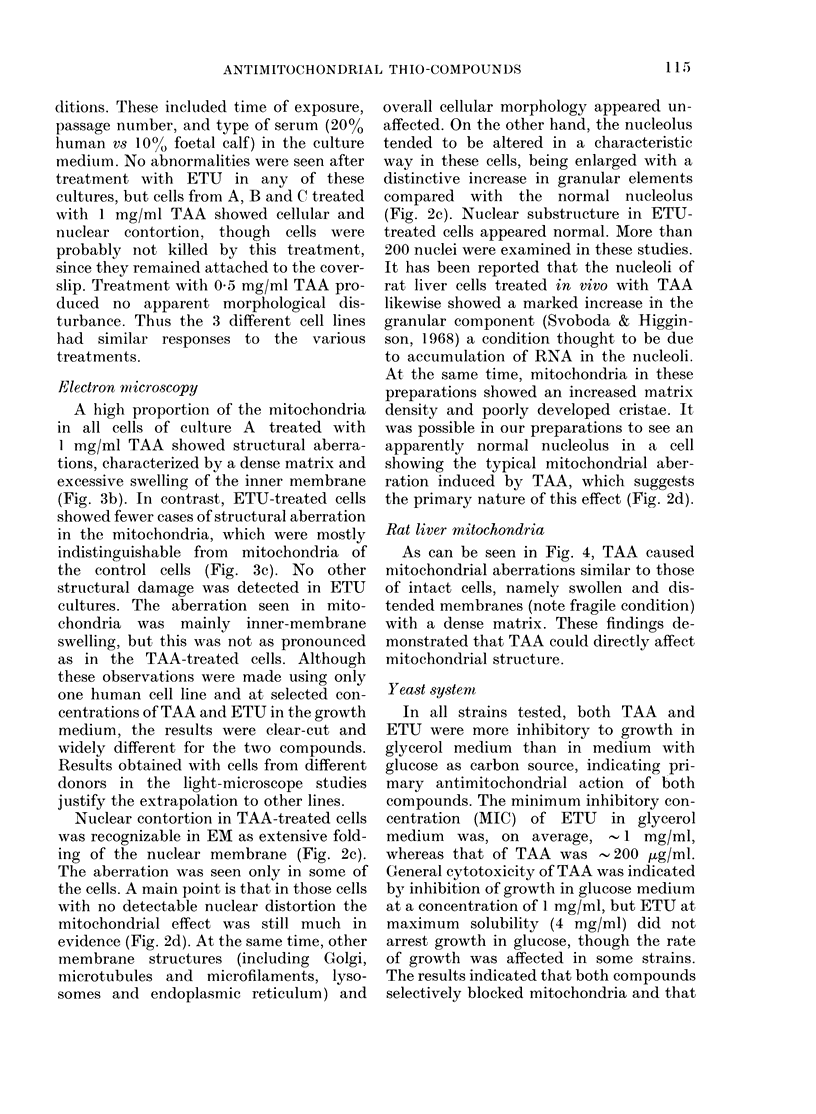

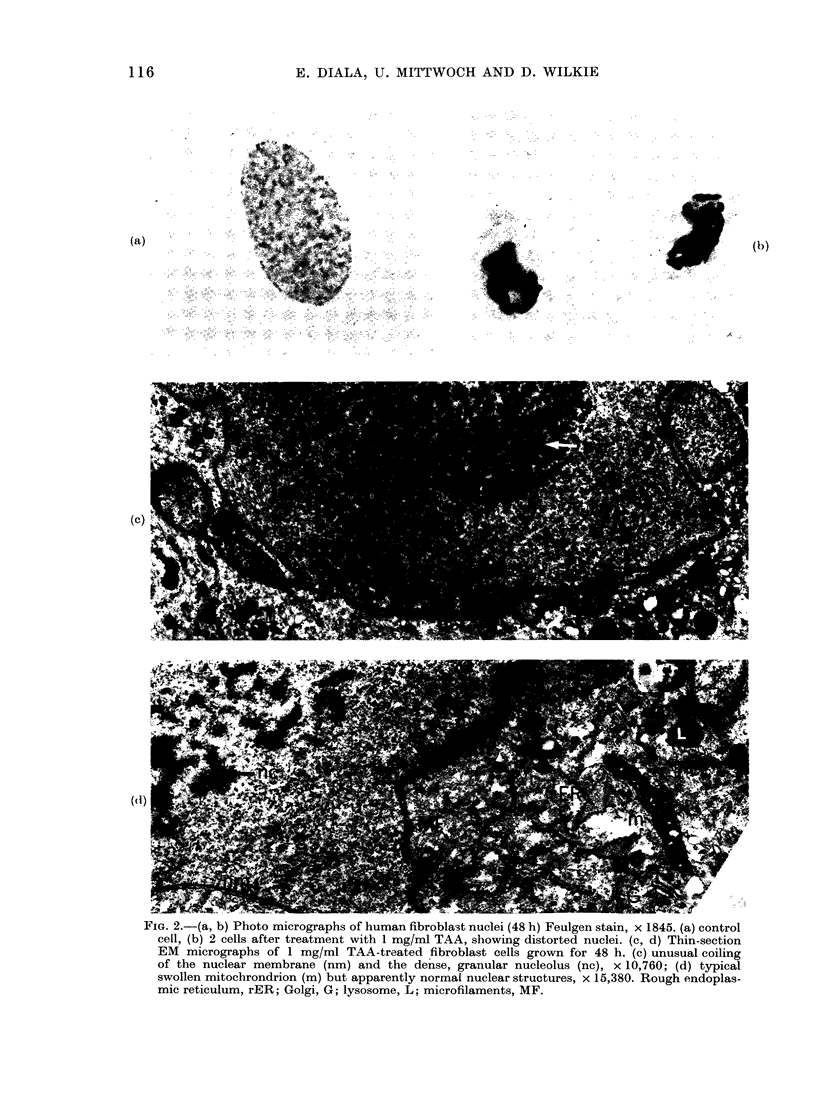

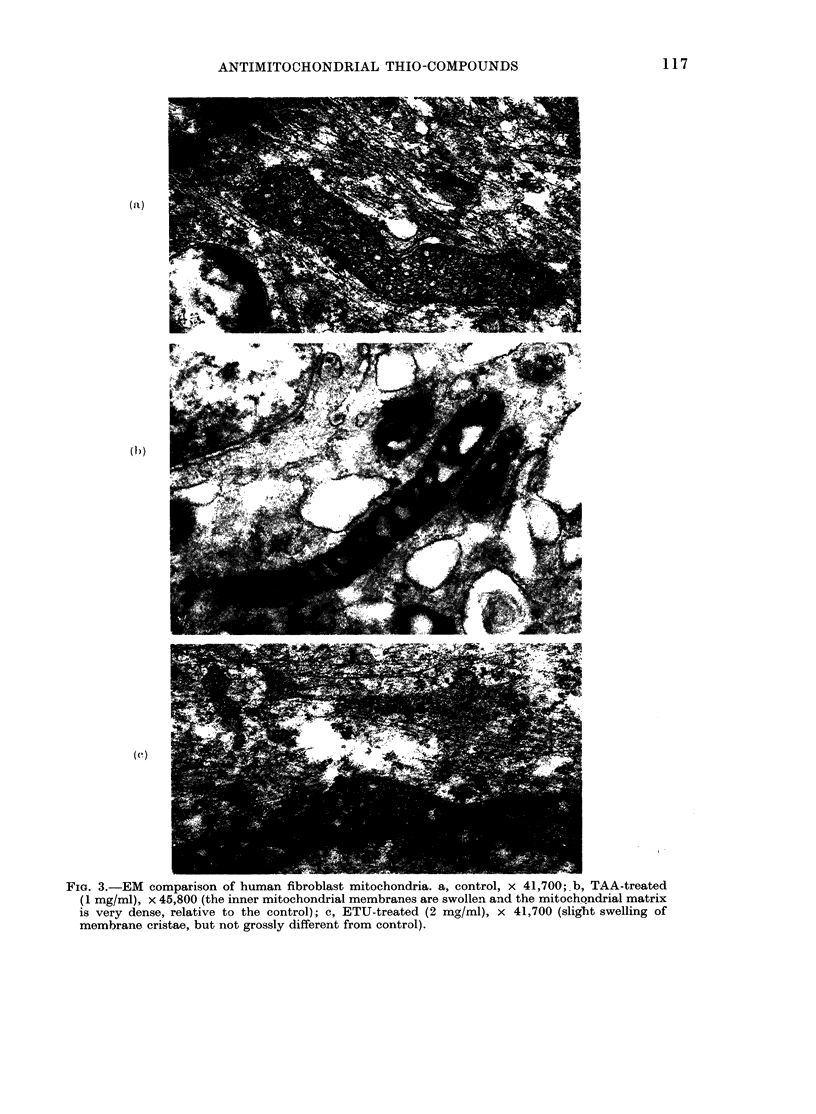

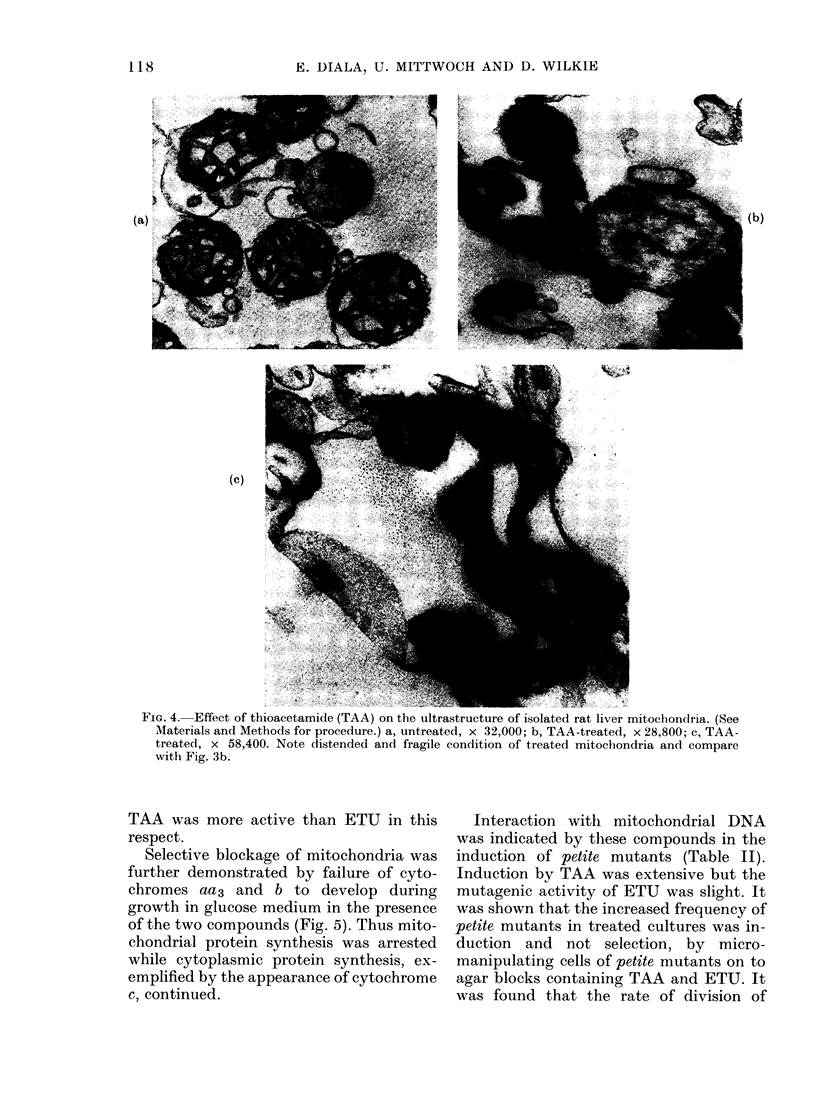

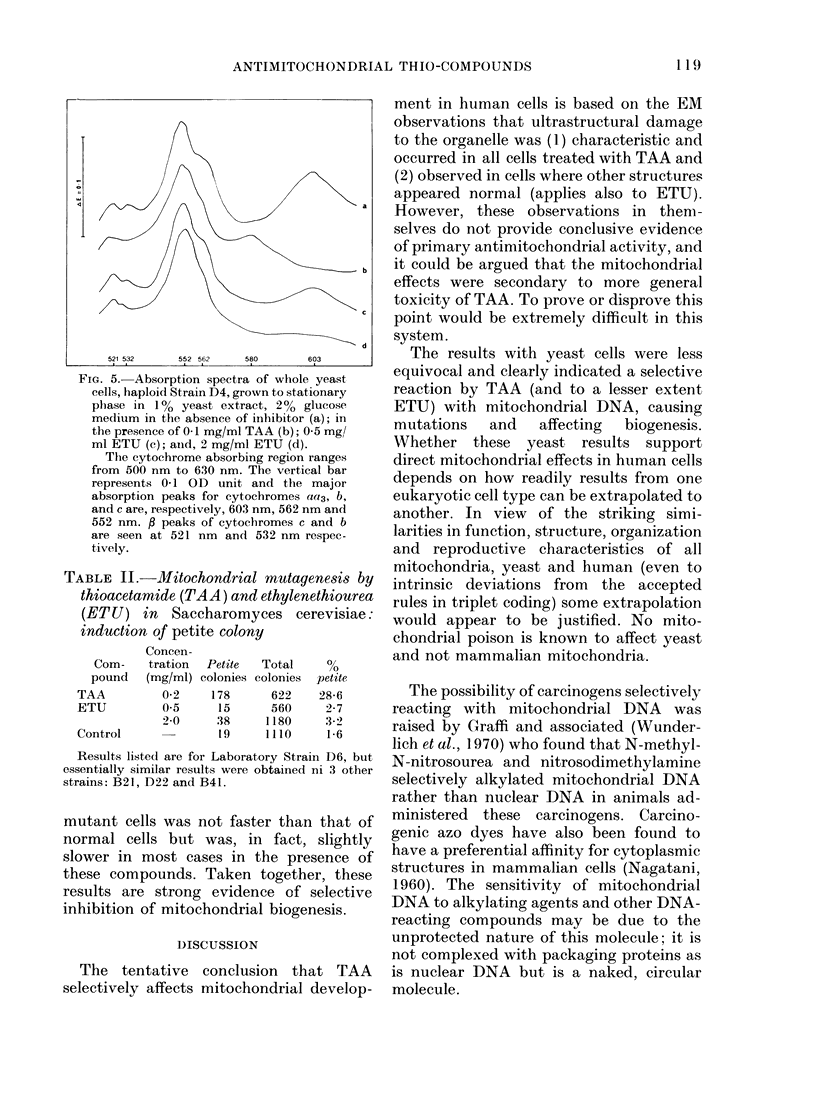

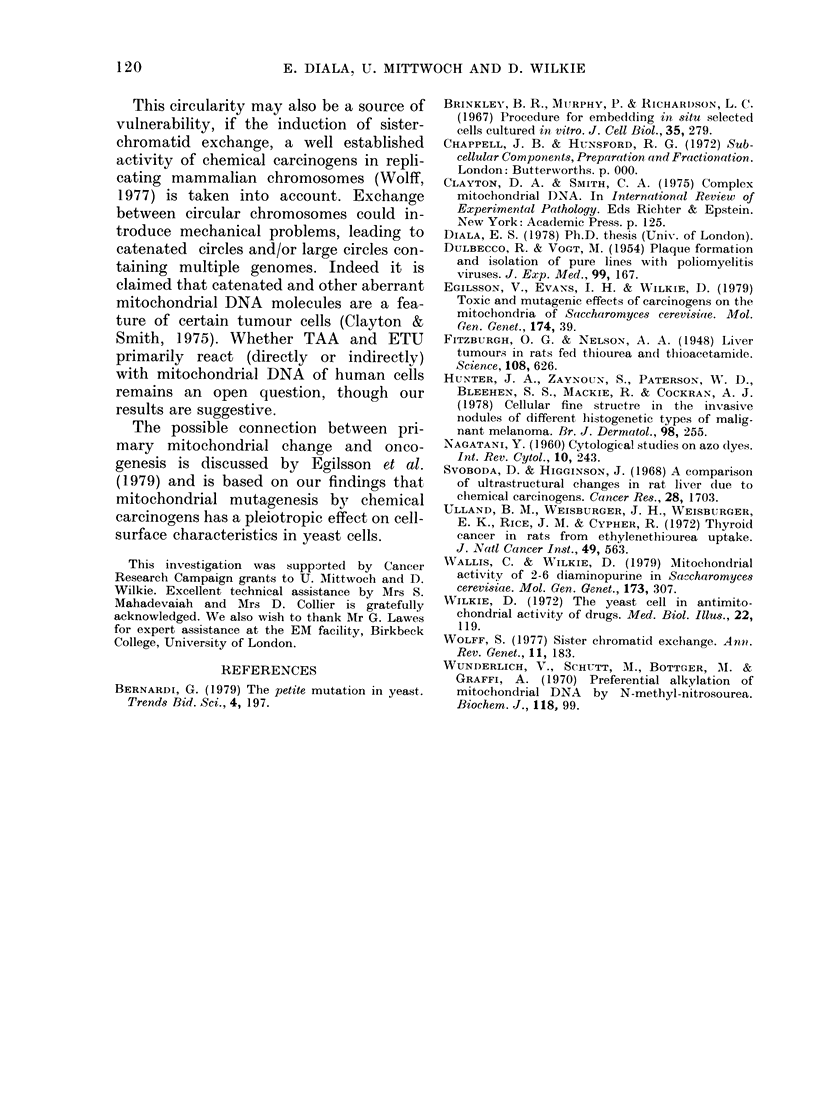


## References

[OCR_00748] Brinkley B. R., Murphy P., Richardson L. C. (1967). Procedure for embedding in situ selected cells cultured in vitro.. J Cell Biol.

[OCR_00765] DULBECCO R., VOGT M. (1954). Plaque formation and isolation of pure lines with poliomyelitis viruses.. J Exp Med.

[OCR_00770] Egilsson V., Evans I. H., Wilkie D. (1979). Toxic and mutagenic effects of carcinogens on the mitochondria of Saccharomyces cerevisiae.. Mol Gen Genet.

[OCR_00776] Fitzhugh O. G., Nelson A. A. (1948). Liver Tumors in Rats Fed Thiourea or Thioacetamide.. Science.

[OCR_00781] Hunter J. A., Zaynoun S., Paterson W. D., Bleehen S. S., Mackie R., Cochran A. J. (1978). Cellular fine structure in the invasive nodules of different histogenetic types of malignant melanoma.. Br J Dermatol.

[OCR_00792] Svoboda D., Higginson J. (1968). A comparison of ultrastructural changes in rat liver due to chemical carcinogens.. Cancer Res.

[OCR_00803] Wallis C., Wilkie D. (1979). Mitochondrial activity of 2,6-diaminopurine in Saccharomyces cerevisiae.. Mol Gen Genet.

[OCR_00808] Wilkie D. (1972). The yeast cell in anti-mitochondrial activity of drugs.. Med Biol Illus.

[OCR_00813] Wolff S. (1977). Sister chromatid exchange.. Annu Rev Genet.

[OCR_00817] Wunderlich V., Schütt M., Böttger M., Graffi A. (1970). Preferential alkylation of mitochondrial deoxyribonucleic acid by N-methyl-N-nitrosourea.. Biochem J.

